# HOXB13 affects the cancer stem cell characteristics of nasopharyngeal carcinoma by regulating the Wnt/β-catenin/SOX2 pathway

**DOI:** 10.1186/s41065-025-00549-7

**Published:** 2025-09-29

**Authors:** Ying Xu, Xia Li

**Affiliations:** Department of Otolaryngology, The Second People’s Hospital of Changzhou, the Third Affiliated Hospital of Nanjing Medical University, No. 68, GeHu Road, Changzhou, 213003 Jiangsu China

**Keywords:** HOXB13, NPC, Wnt/β-catenin/SOX2 pathway, Stem cell, Migration and invasion

## Abstract

**Purpose:**

HOXB13 has been shown to act as a tumor promoter in various malignancies; however, its role in nasopharyngeal carcinoma (NPC) remains unexplored. This study aimed to investigate the function of HOXB13 in NPC and elucidate its underlying mechanism to identify novel targets for NPC diagnosis and therapy.

**Methods:**

HOXB13 expression in NPC was examined through bioinformatic analyses of the TCGA and GEO databases, and the findings were validated using molecular biology techniques. After the transfection of NPC cell lines with siRNA targeting HOXB13 (si-HOXB13), the effects of HOXB13 knockdown on cell proliferation, migration, invasion, and stemness were evaluated. Expression levels of Wnt/β-catenin/SOX2 pathway-related proteins were assessed. In vivo, NPC cells transfected with sh-HOXB13 were injected into nude mice, after which tumor volume and mass were measured, and lung metastases were analyzed using hematoxylin and eosin (H&E) staining.

**Results:**

HOXB13 knockdown significantly reduced NPC cell viability, suppressed clonogenicity and invasiveness, increased scratch width in wound healing assays, and decreased sphere formation and the proportion of CD133^+^ cells. Additionally, si-HOXB13 significantly downregulated the protein expression of β-catenin, c-Myc, and SOX2. In vivo, the sh-HOXB13 group exhibited reduced tumor mass, volume and lung metastatic nodules compared to the sh-NC group.

**Conclusion:**

This study demonstrates that HOXB13 facilitates the malignant progression of NPC by regulating the Wnt/β-catenin/SOX2 signaling pathway, suggesting HOXB13 as a potential therapeutic and diagnostic target for NPC, thereby offering a new strategy to improve patient prognosis.

**Supplementary Information:**

The online version contains supplementary material available at 10.1186/s41065-025-00549-7.

## Introduction

Nasopharyngeal carcinoma (NPC) is a malignant epithelial tumor arising from the nasopharyngeal mucosa [1]. It is characterized by high invasiveness and a propensity for early local infiltration and distant metastasis. Clinically, early-stage NPC is primarily treated with radiotherapy, while patients with advanced disease are typically managed with a combination of chemotherapy and radiotherapy [2]. Although recent advances have significantly improved the five-year survival rate for NPC patients, tumor recurrence and distant metastasis remain the primary causes of treatment failure and mortality [3]. Therefore, elucidating the molecular mechanisms underlying NPC progression and metastasis is essential for identifying novel therapeutic targets and developing more effective treatment strategies.

Homeobox (HOX) genes encode a conserved family of transcription factors defined by the presence of a homeodomain organized into four clusters, namely HOXA, HOXB, HOXC and HOXD, within the human genome. HOXB13, a member of the HOXB cluster, has been implicated in the pathogenesis and progression of various cancers [4]. In cervical cancer, HOXB13 has been shown to activate the NF-κB signaling pathway, thereby promoting tumor growth [5]. In hepatocellular carcinoma (HCC), HOXB13 expression is significantly elevated in tumor tissues compared to adjacent non-tumorous tissues, and its high expression correlates with advanced clinical stage and poor prognosis. Functional studies have demonstrated that HOXB13 overexpression enhances the proliferative and invasive capacities of HCC cells [6]. Similarly, in lung cancer, HOXB13 is markedly overexpressed and is closely associated with tumor aggressiveness and unfavorable clinical outcomes [7].

The Wnt/β-catenin signaling pathway plays an important role in tumor biology, and its aberrant activation has been linked to enhanced proliferation, migration, and maintenance of cancer stem cell properties in various malignancies [8], with accumulating evidence suggesting that HOXB13 may regulate this pathway and thereby influence tumor progression [9].

In this study, we demonstrate for the first time that HOXB13 is significantly overexpressed in NPC tissues, and that its elevated expression is associated with poor clinical prognosis. Functional experiments revealed that HOXB13 knockdown markedly suppresses NPC cell proliferation, migration, invasion, and stemness. Further mechanistic analyses indicated that HOXB13 promotes the malignant phenotype of NPC by regulating the Wnt/β-catenin signaling pathway. These findings suggest that HOXB13 may serve as a novel diagnostic and therapeutic target in NPC.

## Methods

### Biological analysis

The expression level of the HOXB13 gene in head and neck cancer tissues was examined using the UALCAN database. To evaluate the prognostic relevance of HOXB13 expression, survival analysis was performed using the Kaplan-Meier database. Additionally, the expression of HOXB13 in NPC tissues was validated using the GSE53819 gene expression profile dataset retrieved from the GEO database.

### Tissue collection

A total of 30 pairs of NPC tissues and matched adjacent non-tumorous tissues were obtained from patients treated at the Second People’s Hospital of Changzhou. All specimens underwent thorough histopathological review and clinical verification. The study protocol was approved by the hospital’s ethics committee (Approval No. 2025[KY002-01]), and written informed consent was obtained from all participants. After surgical excision, the tissue samples were immediately snap-frozen in liquid nitrogen and stored at -80 °C for further molecular analysis.

### Quantitative PCR (qPCR)

Total RNA was isolated using TRIzol^®^ reagent, and RNA concentration was measured using a NanoDrop spectrophotometer. RNA integrity was verified by 1% agarose gel electrophoresis. Reverse transcription was performed to synthesize cDNA. The real-time quantitative PCR reaction system consisted of SYBR^®^ Green Master Mix, cDNA template, RNase-free H_2_O, and specific forward and reverse primers. Relative mRNA expression levels were calculated using the 2^−ΔΔCt^ method. The primer sequences used for HOXB13 were as follows: Forward, 5′-CCAGTTACCTGGACGTGTCTG-3′; Reverse, 5′-GGACCTGGTGGGTTCTGTTC-3′.

### Immunohistochemistry (IHC)

Formalin-fixed, paraffin-embedded NPC tissue Sect. (4 μm thick) were mounted on anti-stripping glass slides, and the sections were dewaxed in xylene (twice, 10 min each) and rehydrated through a graded ethanol series. To block nonspecific binding, they were incubated with 5% bovine serum albumin (BSA) at room temperature for 30 min and incubated overnight at 4 °C with primary antibody against HOXB13. The next day, an HRP-conjugated secondary antibody was applied and incubated at room temperature for 60 min. Hematoxylin was used for nuclear counterstaining, followed by dehydration with graded ethanol, clearing with xylene, and mounting with neutral gum.

### Cell culture and transfection

The NPC cell line C666-1 was maintained in RPMI-1640 medium supplemented with 10% fetal bovine serum (FBS) and 1% penicillin-streptomycin. The 5–8 F cell line was cultured in Dulbecco’s Modified Eagle Medium (DMEM) containing 10% FBS and 1% penicillin–streptomycin. Following enzymatic digestion and cell counting, the cell density was adjusted to 2 × 10⁵ cells/mL. Cells were seeded into 6-well plates and allowed to adhere and grow for 24 h until approximately 70% confluence was reached. Transfection was performed using Lipofectamine™ 3000 reagent, with two small interfering RNAs targeting HOXB13 (si-HOXB13#1: GCCTCATATTTTCTATCTAGAGC; si-HOXB13#2: ATGATCGTTAGCCTCATATTTTC).

### Cell viability assay (CCK-8)

Cells in the logarithmic growth phase were seeded into 96-well plates at a density of 5 × 10^4^ cells/mL and incubated at 37 °C in a humidified atmosphere with 5% CO_2_ for 24 h. At the indicated time points (0, 24, 48, and 72 h), 10 µL of CCK-8 solution was added to each well. After a 2-hour incubation at 37 °C in the dark, the absorbance at 450 nm was measured using a microplate reader to assess cell viability.

### Colony formation assay

The cells were seeded into 6-well plates at a density of 1,000–2,000 cells per well in 2 mL of complete culture medium, and the plates were incubated at 37 °C with 5% CO_2_ for 14 days, with medium replacement every 2–3 days. Colonies were fixed with 4% paraformaldehyde for 20 min, washed with phosphate-buffered saline (PBS), and stained with 0.1% crystal violet for 30 min. Excess stain was removed by rinsing with running water until the background was clear. The plates were then inverted, dried, and colony formation was observed under a light microscope. Cell clusters containing more than 50 cells were defined as colonies.

### Wound healing assay

Cells were seeded into 6-well plates at a density of 1 × 10^6^ cells per well and cultured to 90–100% confluence. After removing non-adherent cells by gently rinsing with PBS, 1 mL of serum-free medium was added to each well. A sterile pipette tip was used to create a linear scratch across the cell monolayer. The wells were then rinsed with PBS to remove detached cells. Images of three randomly selected fields within the scratch area were captured under a microscope to record the initial wound width. The culture plate then incubated at 37 °C and 5% CO_2_, and the scratch image was taken at the same position 24 h later.

### Transwell

The upper surface of the Transwell membrane (8 μm pore size) was uniformly coated with 50 µL of diluted Matrigel and allowed to solidify at 37 °C. Subsequently, 5 × 10^4^ cells were seeded into the upper chamber in serum-free medium. The lower chamber was filled with 600 µL of complete medium containing 10% FBS as a chemoattractant. The Transwell inserts were incubated at 37 °C in a humidified atmosphere with 5% CO_2_ for 24 h. Following incubation, cells remaining on the upper surface were removed with a cotton swab, and those that had invaded through the membrane were fixed with 4% paraformaldehyde for 20 min and stained with 0.1% crystal violet for 15–20 min. Five random microscopic fields per insert were imaged, and the number of invading cells was quantified using ImageJ software.

### Spheroid formation assay

Cells in the logarithmic growth phase were enzymatically digested and resuspended in complete medium to terminate digestion. After centrifugation, the supernatant was discarded, and cells were washed once with PBS. The pellet was then resuspended in stem cell culture medium consisting of DMEM/F12 supplemented with 20 ng/mL EGF, 20 ng/mL bFGF, B27 supplement, and 1% penicillin–streptomycin. Cell density was adjusted to 5,000 cells/mL. A total of 1000 cells per well were seeded into ultra-low attachment 6-well plates and incubated at 37 °C in a humidified incubator for 7–14 days. Fresh stem cell medium was added every three days, and spheroid formation was monitored every two days using an inverted microscope. Spheres with diameters exceeding 50 μm were considered valid.

### Flow cytometry

Cells were digested with 0.25% trypsin (without EDTA) for two minutes, followed by gentle tapping to detach cells. The digestion was neutralized with serum-containing medium, and the cells were centrifuged at 1000 rpm for five minutes. The supernatant was discarded, and the cells were resuspended in PBS containing 1% bovine serum albumin (BSA) to a final density of 1 × 10^6^ cells/mL. A 100 µL aliquot (approximately 1 × 10^5^ cells) was incubated with PE-conjugated anti-CD133 antibody in the dark for 30 min. After incubation, 2 mL of PBS with 1% BSA was added, followed by centrifugation, and the washing step was repeated once. The final cell suspension was filtered through a 40 μm strainer and resuspended in 300 µL of PBS with 1% BSA. The percentage of CD133-positive cells was analyzed using a flow cytometer.

### Western-blot

Cells or tissues were lysed on ice using RIPA lysis buffer, and total protein concentration was determined by the BCA assay. Equal amounts of protein were subjected to SDS-PAGE, then transferred to a PVDF membrane. The membranes were blocked with 5% skim milk at room temperature for 1 h and incubated overnight at 4 °C with primary antibodies. After washing three times with TBST (10 min each), membranes were incubated with HRP-conjugated secondary antibodies at room temperature for 1 h. Following another three TBST washes, immunoreactive bands were visualized using a chemiluminescent imaging system. Band intensities were quantified using appropriate imaging software. The primary antibodies used were as follows: HOXB13 (Abcam, ab201682, 1:5000), β-catenin (Affinity, AF6266, 1:1000), SOX2 (Affinity, AF5140, 1:1000), and c-Myc (Affinity, AF6054, 1:1000).

### Xenograft tumor experiments

Male BALB/c nude mice (18–20 g, aged 4–6 weeks) were maintained under specific pathogen-free (SPF) conditions. A total of 12 mice were randomly divided into two groups (*n* = 6 per group): sh-NC and sh-HOXB13. Each mouse was subcutaneously injected with 100 µL of a single-cell suspension containing 1 × 10^6^ NPC cells into the right axillary region. After injection, the puncture site was gently compressed to prevent leakage. Tumor volumes were measured every five days using calipers, and calculated using the formula: volume = (length × width^2^)/2. At the end of the experiment, the mice were euthanized via cervical dislocation, and tumors were excised, measured, and weighed. Tumor growth curves were plotted with time on the x-axis and tumor volume on the y-axis.

### Hematoxylin and Eosin (H&E) staining

NPC tumor tissues fixed in formalin and embedded in paraffin were sectioned into 4 μm slices using a rotary microtome. The staining procedure followed the same initial steps as immunohistochemistry. The tissue sections were then counterstained with hematoxylin, dehydrated through a graded ethanol series, cleared with xylene, and mounted with neutral resin. Five microscopic fields were randomly selected from each section to evaluate and count the number of metastatic foci. All animal procedures were reviewed and approved by the Animal Ethics Committee of the Third Affiliated Hospital of Nanjing Medical University (Approval No. 2025[KY002-01]).

### Statistical analysis

All statistical analyses were performed using SPSS software. Each experiment was repeated independently at least three times. Data are presented as mean ± standard deviation (SD). Comparisons between two groups were conducted using the two-tailed Student’s t-test, while differences among multiple groups were assessed using one-way analysis of variance (ANOVA). A *P*-value < 0.05 was considered statistically significant.

## Results

### High expression of HOXB13 in NPC and its correlation with prognosis

**Fig. 1:**
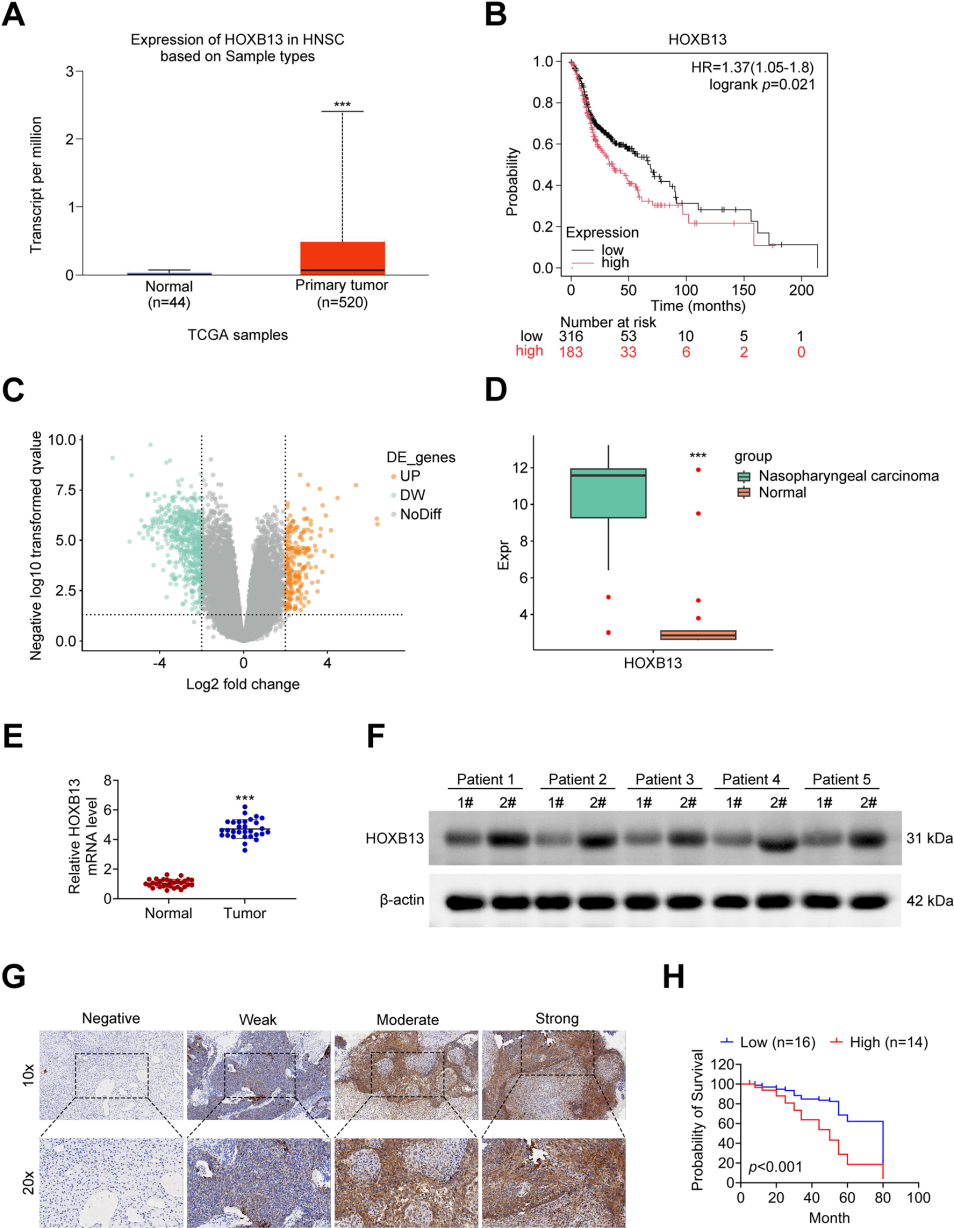
High levels of HOXB13 are observed in NPC. (**A**) Expression of HOXB13 in tumor and normal tissues from patients with head and neck cancer, based on data from the UALCAN database. (**B**) Kaplan–Meier analysis of overall survival in head and neck cancer patients stratified by HOXB13 expression. (**C**) Volcano plot of differentially expressed genes in the GSE53819 dataset. (**D**) HOXB13 expression levels in NPC tissues, as presented in the GSE53819 expression profile. (**E**) Quantitative PCR analysis of HOXB13 mRNA expression in 30 paired NPC and adjacent normal tissues. (**F**) Western blot analysis of HOXB13 protein levels in the same cohort. (**G**) Immunohistochemical detection of HOXB13 expression in NPC tissue samples. (**H**) Survival curve of NPC patients. ^***^ *P < 0.001 vs. Normal*

Given that NPC is classified under head and neck cancers, the expression level of HOXB13 in head and neck cancer was initially examined using the TCGA database, which revealed significantly higher HOXB13 expression in tumor tissues compared to normal tissues (Fig. 1A). Kaplan-Meier survival analysis indicated that patients with high HOXB13 expression exhibited a lower overall survival rate than those with low expression levels (Fig. 1B). To further validate these findings in NPC, differential gene expression analysis was performed using the GSE53819 dataset from the GEO database, with logFC = 2 and Padj < 0.05 set as cutoff criteria. The volcano plot illustrated the distribution of differentially expressed genes (Fig. 1C), and HOXB13 was among the significantly upregulated genes in NPC tissues (Fig. 1D). Additionally, tumor and matched adjacent non-tumorous tissues were collected from 30 patients diagnosed with NPC. Quantitative PCR and Western blot analyses were performed to assess HOXB13 expression at the mRNA and protein levels, respectively, and demonstrated that HOXB13 mRNA expression was significantly elevated in NPC tissues compared to adjacent tissues (Fig. 1E), consistent with the increase observed in protein expression (Fig. 1F). Immunohistochemical staining of NPC tumor tissues further confirmed HOXB13 overexpression (Fig. 1G). Based on staining intensity, patients were stratified into high and low HOXB13 expression groups. Survival analysis revealed that patients with high HOXB13 expression had significantly poorer overall survival compared to those with low expression (Fig. 1H).

### HOXB13 knockdown inhibits the proliferation of NPC cells

**Fig. 2:**
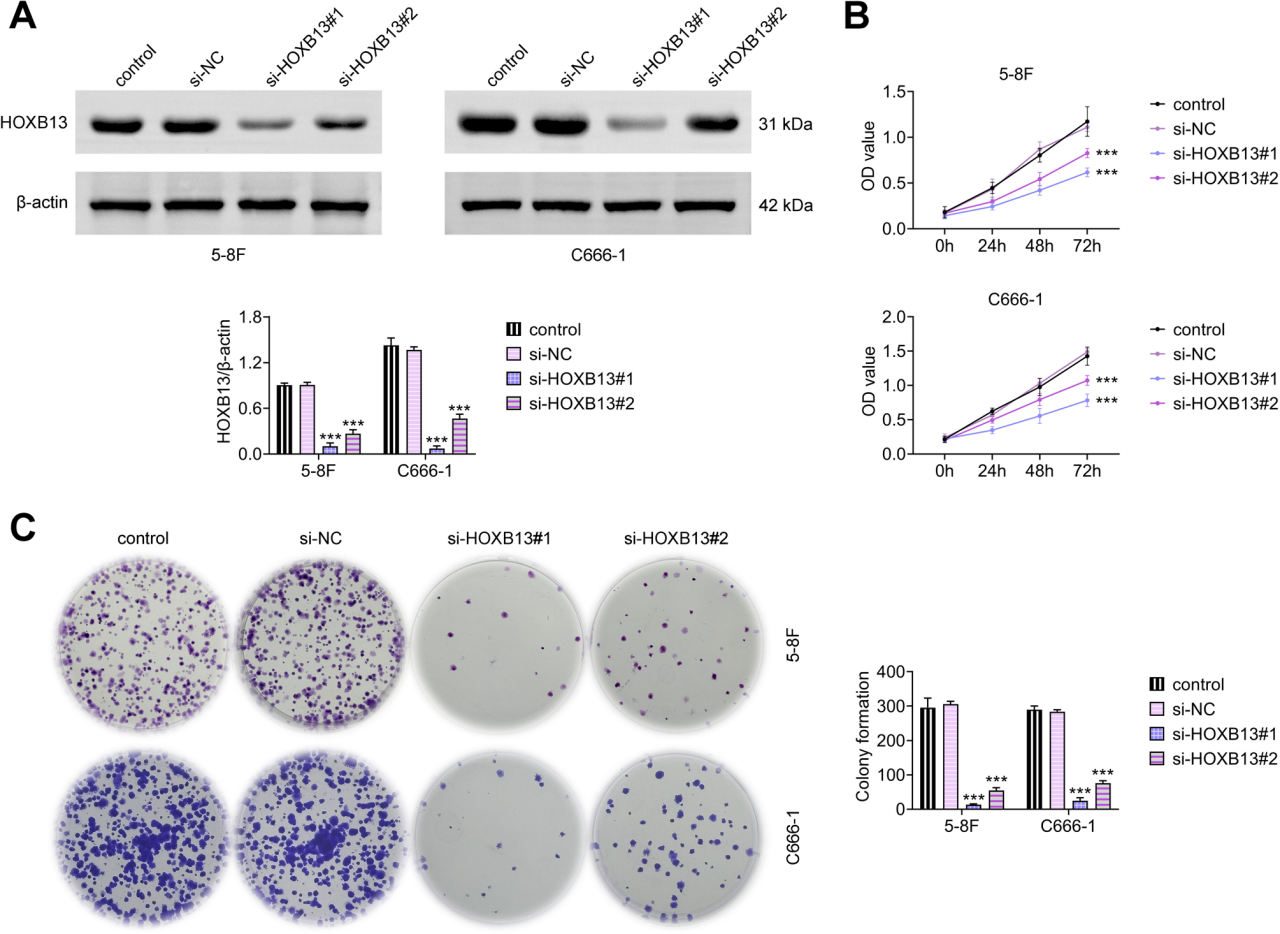
HOXB13 knockdown suppresses NPC cell proliferation. (**A**) Western blot analysis of HOXB13 protein levels following knockdown in NPC cells. (**B**) CCK-8 assay to assess cell viability at multiple time points. (**C**) Colony formation assay to quantify proliferative capacity. ^***^ *P < 0.001 vs. control*

Western blot analysis was performed to evaluate the transfection efficiency of si-HOXB13 in NPC cell lines 5–8 F and C666-1. Among the two constructs, si-HOXB13#1 demonstrated stronger silencing efficiency compared to si-HOXB13 #2 (Fig. 2A), and CCK-8 assay (Fig. 2B) and colony formation assay (Fig. 2C) revealed that knockdown of HOXB13 significantly reduced cell viability and the number of crystal violet-stained colonies in both cell lines. Notably, si-HOXB13#1 exerted a more pronounced inhibitory effect on NPC cell proliferation.

### HOXB13 knockdown inhibits the migration and invasion of NPC cells

**Fig. 3:**
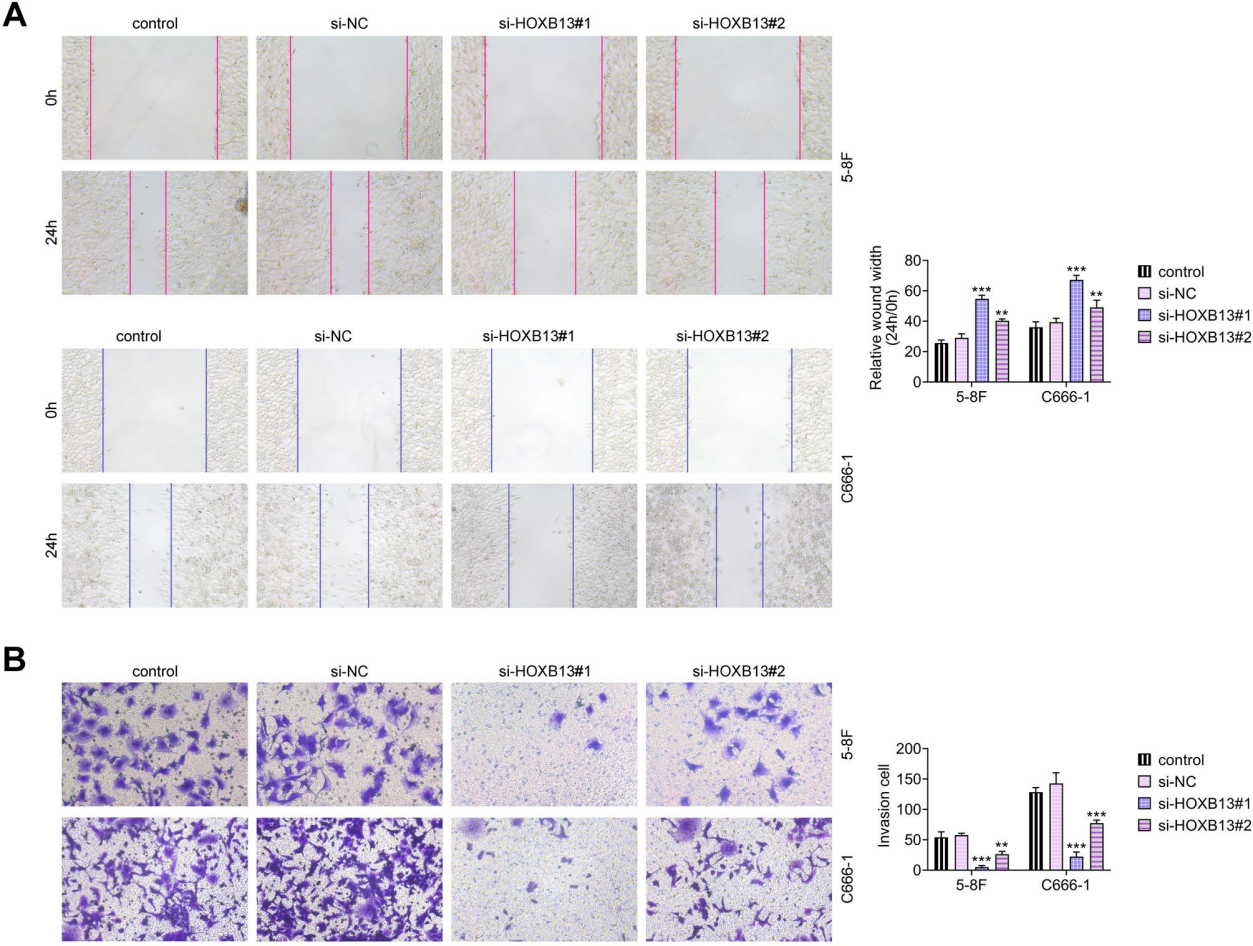
HOXB13 knockdown reduces NPC cell migration and invasion. (**A**) Wound healing assay to evaluate cell migration. (**B**) Transwell invasion assay to assess invasive capability. ^**^ *P* < 0.01, ^***^ *P < 0.001 vs. control*

To assess the effects of HOXB13 knockdown on cellular motility, wound healing and Transwell assays were performed. The scratch assay demonstrated that HOXB13 knockdown markedly reduced the migratory capacity of NPC cells (Fig. 3A). Similarly, the number of invasive cells detected in the Transwell assay was significantly decreased following transfection with si-HOXB13 (Fig. 3B). In both assays, si-HOXB13#1 produced a stronger inhibitory effect than si-HOXB13#2. These results indicate that HOXB13 knockdown suppresses both the migration and invasion of NPC cells.

### HOXB13 knockdown inhibits the stemness of NPC cells

**Fig. 4:**
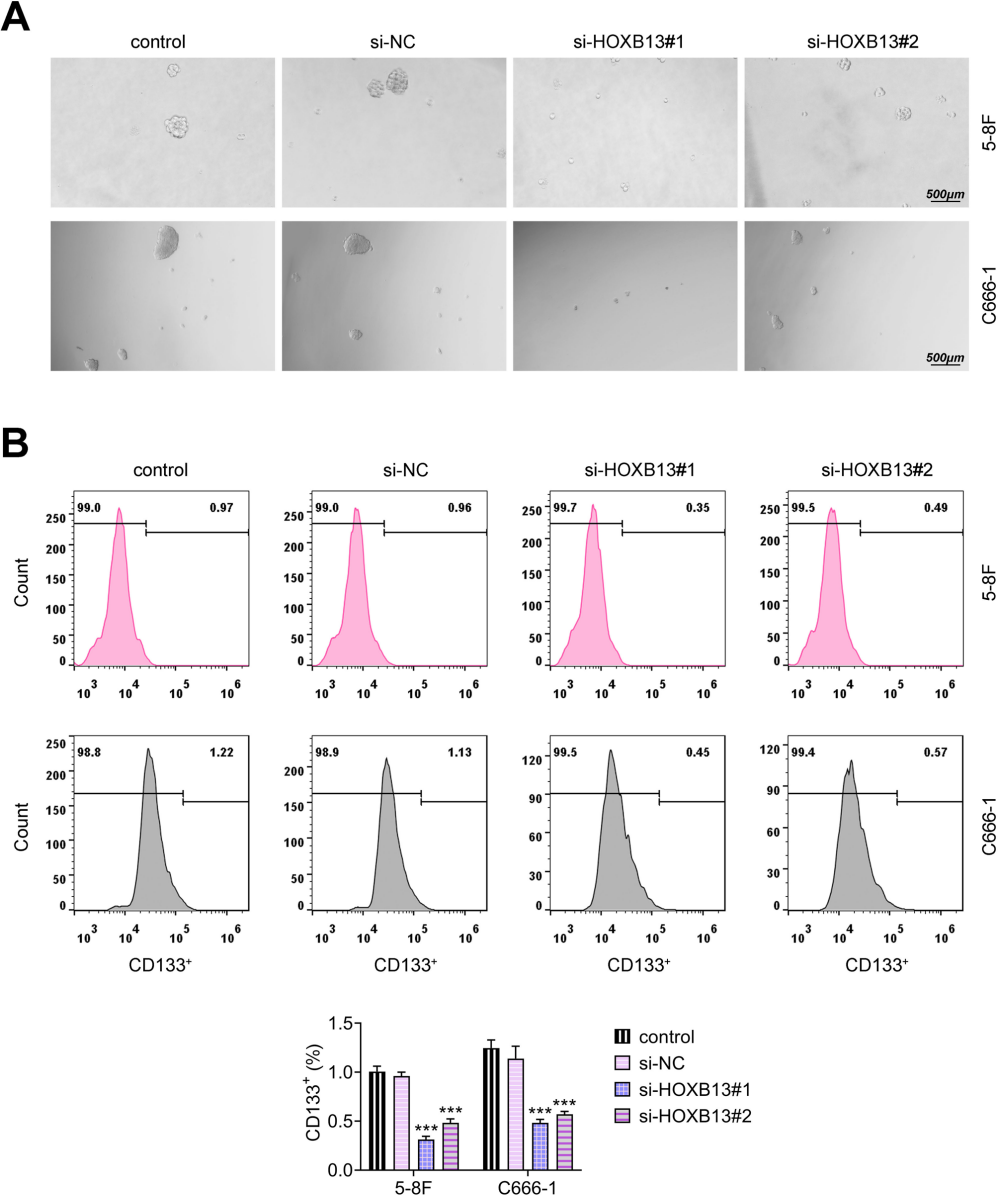
HOXB13 knockdown diminishes the stemness of NPC cells. (**A**) Spheroid formation assay to assess the self-renewal capacity of NPC cells. (**B**) Flow cytometric analysis of the CD133^+^ subpopulation in NPC cells. ^***^ *P < 0.001 vs. control*

To investigate the impact of HOXB13 on the stem-like properties of NPC cells, spheroid formation and flow cytometry analyses were conducted and revealed that HOXB13 knockdown significantly reduced the number of tumor spheres generated (Fig. 4A). Furthermore, flow cytometry analysis indicated a decrease in the proportion of CD133^+^ cells, a commonly used marker of cancer stemness, in the si-HOXB13-transfected cells (Fig. 4B). These findings suggest that HOXB13 knockdown impairs the stemness characteristics of NPC cells.

### The Wnt/β-catenin/SOX2 pathway is inhibited by HOXB13 knockdown

**Fig. 5:**
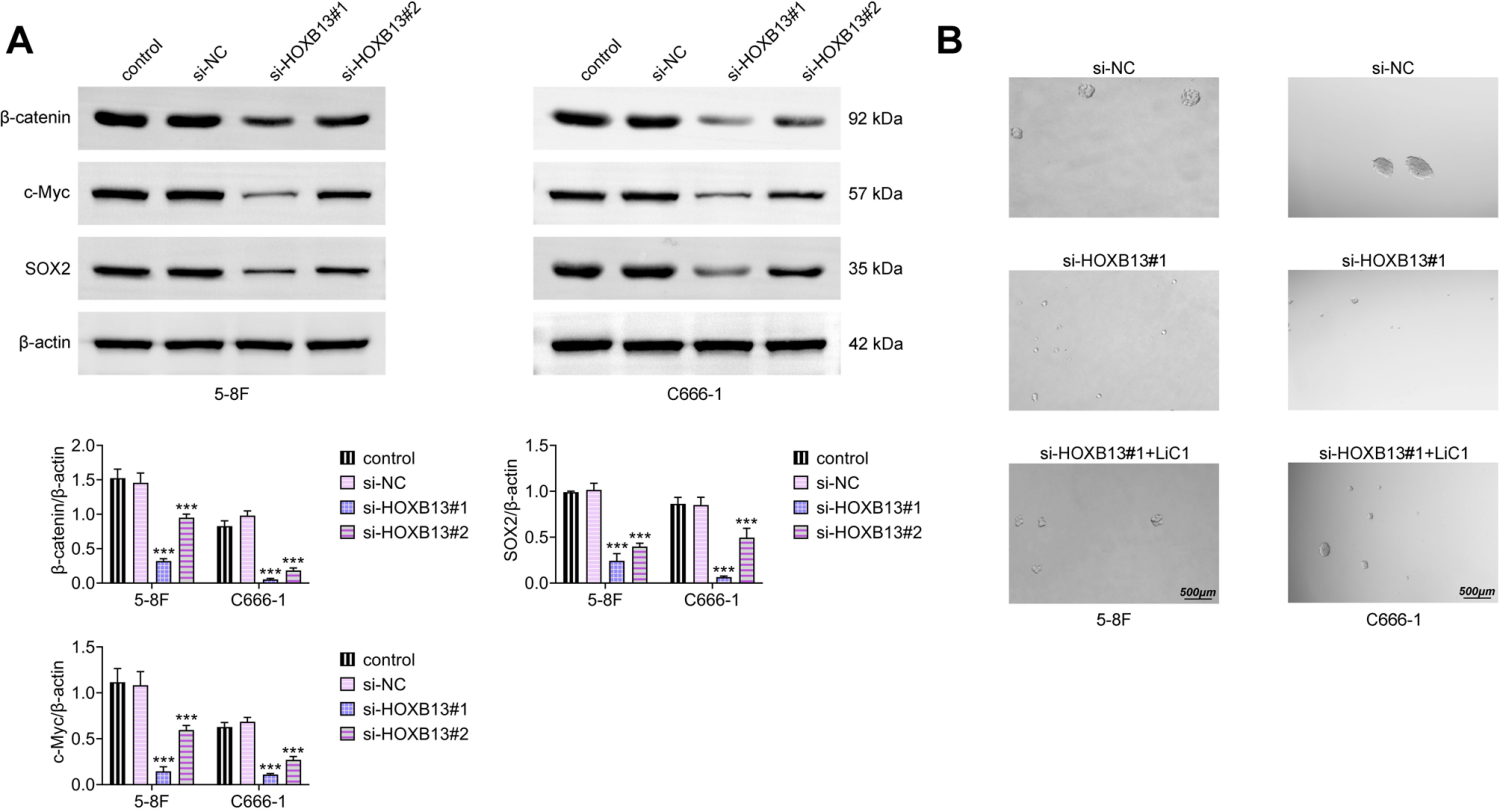
HOXB13 knockdown inhibits the Wnt/β-catenin/SOX2 signaling pathway. (**A**) Western blot analysis of β-catenin, c-Myc, and SOX2 protein expression in NPC cells. (**B**) Spheroid formation assay to examine changes in stem-like properties. ^***^ *P < 0.001 vs. control*

Westernblot analysis showed that HOXB13 knockdown significantly decreased the expression of of β-catenin, c-Myc, and SOX2, with si-HOXB13#1 producing a greater suppressive effect (Fig. 5A). To further validate the involvement of this pathway, a rescue experiment was conducted using LiCl, a known activator of Wnt signaling [10]. Treatment with Wnt3a successfully reversed the suppression of NPC cell stemness induced by HOXB13 knockdown (Fig. 5B), thereby confirming that HOXB13 exerts its effects, at least in part, through regulation of the Wnt/β-catenin/SOX2 pathway.

### Knockdown of HOXB13 inhibits tumor growth and metastasis in vivo

**Fig. 6:**
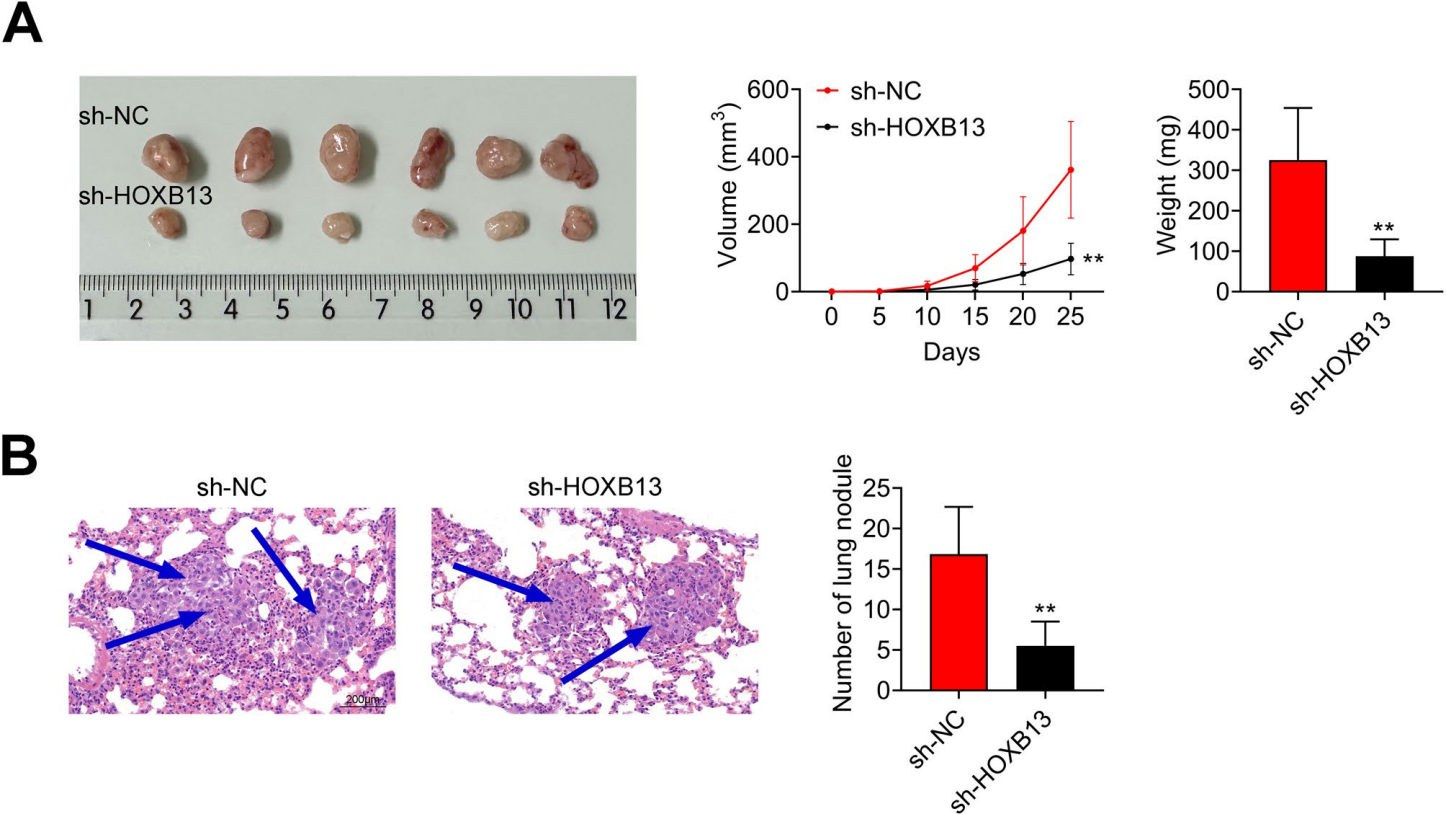
HOXB13 knockdown suppresses tumor growth and metastasis in vivo. (**A**) Tumor size, volume, and weight in xenograft models from mice injected with sh-NC or sh-HOXB13-transfected NPC cells. (**B**) H&E staining of lung tissues to assess metastatic foci. ^**^ *P < 0.01 vs. sh-NC*

To evaluate the effects of HOXB13 knockdown on tumor growth and metastasis in vivo, NPC cells transfected with either sh-NC or sh-HOXB13 were subcutaneously injected into nude mice. Tumor development was monitored, and the results showed that both tumor volume and tumor weight were significantly reduced in the sh-HOXB13 group compared to the sh-NC group (Fig. 6A). In addition, H&E staining of lung tissues revealed metastatic tumor foci in both groups; however, the number of lung metastases was markedly lower in the sh-HOXB13 group (Fig. 6B). These results indicate that HOXB13 knockdown effectively suppresses NPC tumor growth and distant metastasis in vivo.

## Discussion

Despite significant advances in multimodal comprehensive treatment that have markedly improved the local control rate of NPC, local recurrence and distant metastasis remain the primary causes of treatment failure and mortality. This persistent clinical challenge underscores the importance of in-depth investigation into the molecular mechanisms underlying NPC metastasis, which is essential for establishing a theoretical framework to support the development of new therapeutic strategies [11].

Cancer stem cells (CSCs), a distinct subpopulation of tumor cells characterized by self-renewal capacity and the potential for multidirectional differentiation, play a pivotal role in the metastatic progression of tumors. Also referred to as tumor-initiating cells, CSCs have garnered substantial research interest due to their unique biological properties. Notably, CSCs exhibit pronounced resistance to conventional chemotherapy and radiotherapy, contributing to suboptimal clinical outcomes and serving as a major source of tumor recurrence and distant spread [12]. Based on this understanding, current investigations have increasingly focused on identifying the molecular markers and regulatory pathways specific to CSCs to establish precise detection techniques and designing targeted therapeutic interventions for this cellular subset.

Tumor cell proliferation is an important process tumor growth and progression. Previous studies have demonstrated that HOXB13 promotes proliferation in several types of malignancies by modulating genes associated with the cell cycle [13]. Consistent with these findings, our data suggest that HOXB13 plays a similar role in NPC. Specifically, silencing HOXB13 expression markedly suppresses NPC cell proliferation, thereby reinforcing its potential as a promising therapeutic target in the management of this disease.

Tumor cell migration and invasion are key processes in the metastatic cascade of malignancies [14]. Previous studies have shown that HOXB13 promotes these behaviors in various cancers by regulating genes involved in the epithelial–mesenchymal transition (EMT) [15]. Consistent with these findings, our results indicate that HOXB13 contributes to NPC progression through a similar mechanism. Silencing HOXB13 significantly reduced NPC cell migration and invasion, further supporting its potential as a therapeutic target.

The initiation and progression of NPC are closely regulated by the Wnt/β-catenin signaling pathway, whose dysregulation is known to enhance tumor cell proliferation, migration, and malignant transformation. Beyond its role in promoting invasion and metastasis, aberrant activation of Wnt/β-catenin also facilitates tumor initiation and sustains cancer stem cell properties in NPC [16]. Importantly, Wnt/β-catenin signaling maintains cellular stemness in part through the regulation of SOX2 expression in cancer cells [17]. Essential stemness factors such as SOX2 and c-Myc are essential for preserving stem cell characteristics and promoting proliferation, thereby influencing the biological behavior of CSCs [18]. SOX2 has been recognized as an oncogenic factor capable of enhancing tumor cell growth and dissemination [19]. Similarly, c-Myc, an important transcription factor aberrantly expressed in various human malignancies, regulates a broad spectrum of genes involved in angiogenesis, metabolism, apoptosis, stemness maintenance, and proliferation, thereby contributing to tumor progression [20]. In the present study, knockdown of HOXB13 not only suppressed NPC cell proliferation, migration, invasion, and stemness, but also reduced the expression levels of β-catenin, SOX2, and c-Myc, suggesting that HOXB13 may exert its oncogenic effects through this regulatory axis.

Several limitations of the current study should be acknowledged. First, rescue experiments were not performed to confirm the specific role of HOXB13 in regulating the Wnt/β-catenin/SOX2 pathway. Second, further experimental validation is required to clarify whether the anti-tumor effects of si-HOXB13 are indeed mediated through this signaling cascade. Third, considering the established link between tumor cell stemness and therapeutic resistance, future investigations are warranted to explore whether targeting HOXB13 could modulate resistance mechanisms in NPC. Despite these limitations, our findings highlight HOXB13 as a potential molecular target for therapeutic strategies to impair stemness and metastasis in NPC.

## Supplementary Information

Below is the link to the electronic supplementary material.

Supplementary Material 1

## Data Availability

No datasets were generated or analysed during the current study.
