# Supplementary material for: HOXB13 affects the cancer stem cell characteristics of nasopharyngeal carcinoma by regulating the Wnt/β-catenin/SOX2 pathway
Source: Hereditas. 2025 Sep 29;162:192. doi: 10.1186/s41065-025-00549-7 (PMC12482284; doi:10.1186/s41065-025-00549-7)
Supplement: Supplementary file 1 — Supplementary Material 1 [file 41065_2025_549_MOESM1_ESM.docx]

Fig1-f

1. HOXB13

实验分组: (1) Patient 1 1# 2# (2) Patient 2 1# 2# (3) Patient 3 1# 2# (4) Patient 4 1# 2# (5) Patient 5 1# 2#





2. β-actin

实验分组: (1) Patient 1 1# 2# (2) Patient 2 1# 2# (3) Patient 3 1# 2# (4) Patient 4 1# 2# (5) Patient 5 1# 2#





Fig2-a

5-8F

1. HOXB13

实验分组：（1）control（2）si-NC（3）si-HOXB13#1（4）si-HOXB13#2





2. β-actin

实验分组：（1）control（2）si-NC（3）si-HOXB13#1（4）si-HOXB13#2





C666-1

1. HOXB13

实验分组：（1）control（2）si-NC（3）si-HOXB13#1（4）si-HOXB13#2





2. β-actin

实验分组：（1）control（2）si-NC（3）si-HOXB13#1（4）si-HOXB13#2





Fig5-wb

5-8F

1. β-catenin

实验分组：（1）control（2）si-NC（3）si-HOXB13#1（4）si-HOXB13#2





2. c-Myc

实验分组：（1）control（2）si-NC（3）si-HOXB13#1（4）si-HOXB13#2





3. SOX2

实验分组：（1）control（2）si-NC（3）si-HOXB13#1（4）si-HOXB13#2





4. β-actin

实验分组：（1）control（2）si-NC（3）si-HOXB13#1（4）si-HOXB13#2





C666-1

1. β-catenin

实验分组：（1）control（2）si-NC（3）si-HOXB13#1（4）si-HOXB13#2





2. c-Myc

实验分组：（1）control（2）si-NC（3）si-HOXB13#1（4）si-HOXB13#2





3. SOX2

实验分组：（1）control（2）si-NC（3）si-HOXB13#1（4）si-HOXB13#2





4. β-actin

实验分组：（1）control（2）si-NC（3）si-HOXB13#1（4）si-HOXB13#2
